# Oxidative Stress and Lung Fibrosis: Towards an Adverse Outcome Pathway

**DOI:** 10.3390/ijms241512490

**Published:** 2023-08-06

**Authors:** Patrudu Makena, Tatiana Kikalova, Gaddamanugu L. Prasad, Sarah A. Baxter

**Affiliations:** 1 RAI Services Company, P.O. Box 1487, Winston-Salem, NC 27102, USA; baxters@rjrt.com; 2 Clarivate Analytics, 1500 Spring Garden, Philadelphia, PA 19130, USA; 3 Former Employee of RAI Services Company, Winston-Salem, NC 27101, USA; 4 Prasad Scientific Consulting LLC, 490 Friendship Place Ct, Lewisville, NC 27023, USA

**Keywords:** cigarette smoking, lung fibrosis, oxidative stress, inflammation, reactive oxygen species, AOP

## Abstract

Lung fibrosis is a progressive fatal disease in which deregulated wound healing of lung epithelial cells drives progressive fibrotic changes. Persistent lung injury due to oxidative stress and chronic inflammation are central features of lung fibrosis. Chronic cigarette smoking causes oxidative stress and is a major risk factor for lung fibrosis. The objective of this manuscript is to develop an adverse outcome pathway (AOP) that serves as a framework for investigation of the mechanisms of lung fibrosis due to lung injury caused by inhaled toxicants, including cigarette smoke. Based on the weight of evidence, oxidative stress is proposed as a molecular initiating event (MIE) which leads to increased secretion of proinflammatory and profibrotic mediators (key event 1 (KE1)). At the cellular level, these proinflammatory signals induce the recruitment of inflammatory cells (KE2), which in turn, increase fibroblast proliferation and myofibroblast differentiation (KE3). At the tissue level, an increase in extracellular matrix deposition (KE4) subsequently culminates in lung fibrosis, the adverse outcome. We have also defined a new KE relationship between the MIE and KE3. This AOP provides a mechanistic platform to understand and evaluate how persistent oxidative stress from lung injury may develop into lung fibrosis.

## 1. Introduction

Lung epithelium is exposed to a variety of insults, including environmental pollutants, xenobiotics, and microbial infections, which cause lung injury [1,2]. Normal lung function and homeostasis are maintained through tightly regulated repair processes. However, under certain conditions, such as chronic cigarette smoking and sustained exposure to environmental toxins, cellular injuries may persist; such repeated injuries could deregulate tissue repair, leading to chronic inflammation and altered tissue architecture, as observed in lung fibrosis. 

Fibrosis is defined by the overgrowth, hardening, and scarring of various tissues and is attributed to excessive deposition of extracellular matrix (ECM) components in the basement membrane and interstitial tissue due to the expansion of activated mesenchymal cells (myofibroblasts). Fibrosis compromises the lung’s ability to perform the gas exchange and is a comorbidity in COPD, lung cancer, and in COVID-19 infections [3,4]. Idiopathic pulmonary fibrosis (IPF) is a serious disease of unknown etiology in which massive fibrotic changes occur in the lung, culminating in respiratory failure [5]. IPF is a progressive, aggressive disease diagnosed usually in the later years of life and has environmental and genetic susceptibility as risk factors [6,7,8]. The prevalence of IPF increases with older age, male gender and cigarette smoking. Smokers are at increased risk of developing IPF and have a shorter survival relative to nonsmokers [9].

Cigarette smoking is a major preventable risk factor for lung fibrosis, COPD, lung cancer and cardiovascular diseases which carry a high degree of mortality and morbidity [10]. Cigarette smoke (CS) is a complex dynamic aerosol consisting of over 8,500 chemicals and toxicants that exist in particulate and volatile gas vapor phases [11]. CS is rich in highly reactive oxygen species (ROS) and toxicants that generate ROS through redox cycling. ROS include reactive oxygen and nitrogen species, which cause oxidative stress [10,12].

Among the key mechanisms that cause smoking-related diseases and those caused by other inhaled toxins and microbial infections, oxidative stress and inflammation are some of the early events that drive additional cellular mechanisms. Oxidative stress is generally defined as an overall imbalance in the cellular/tissue oxidants and anti-oxidants (reductants). While under normal conditions, cellular/tissue anti-oxidant defenses maintain a physiological redox balance caused by injury or infections, chronic insults such as cigarette smoking overwhelm the cellular/tissue antioxidant resources, leading to oxidative stress [13,14]. Normal redox cycling is an important physiological process compartmentalized to the tissue microenvironment and differs from oxidative stress. For example, NADPH oxidases generate ROS and serve essential functions in mediating host defenses against pathogens and in redox-based cell signaling [15,16]. However, under pathological conditions, these intracellular signaling mechanisms are deregulated, leading to oxidative stress.

Considering that CS contains several classes of toxicants and ROS, oxidative stress is persistently present in smokers. While inflammation is a deregulated central process in lung fibrosis [8,17,18,19]; oxidative stress is also hypothesized as a key force in the pathophysiology of the disease [20,21]. Because numerous deregulated cellular and molecular processes drive the progression to fibrosis, a simpler and common approach to interrogate underlying mechanisms of lung fibrosis would be of value.

Adverse outcome pathways (AOPs) are conceptual frameworks used to enhance the utility of pathway-based data for assessing hazards by leveraging non-animal testing methods, (e.g., in vitro assays and in silico methods) [22]. An AOP is a modular theoretical framework that describes a series of mechanistic events by which substances (termed as stressors), can cause adverse human health effects. Such frameworks, comprised of pre-clinical, clinical, and population studies, can be used to predict adverse effects for regulatory decision-making and protect human health using non-animal approaches [23]. 

Briefly, AOPs share a common structure consisting of a molecular initiating event (MIE), a series of key events (KEs) connected by key event relationships (KERs) at molecular, cellular, tissue and organ levels and an adverse outcome (AO). By definition, a KE represents measurable change in a biological state that is essential, but not necessarily sufficient for progression from MIE to AO. The MIE and AO are specialized KE types. AOPs are agnostic to stressors, but are specific to MIEs, and allow the use of existing KEs. KERs define causal relationships between two KEs and establish a sequential relationship. Further, the AOPs are living documents and are updated as new knowledge is acquired [24]. 

Several AOPs for lung fibrosis are at various stages of development [25]. Since oxidative stress is an important early trigger that deregulates numerous profibrotic pathways [26,27,28,29,30,31], we propose an encompassing AOP for lung fibrosis with oxidative stress as an MIE. There are several in-depth authoritative reviews on lung fibrosis and the pathophysiological effects of smoking; hence, we focus on oxidative stress-related aspects of this framework, which serves as a putative AOP. 

Given that CS is abundant in toxicants and carcinogens, and that smoking is the source for persistent oxidative stress and a major risk factor for lung fibrosis, we have developed a putative AOP for inhaled toxicants with cigarette smoking as a representative chemical stressor [26,27,28,29,30,31]. This encompassing AOP for lung fibrosis organizes extensive scientific knowledge into a simple linear format and is anticipated to facilitate the development of fit-for-purpose alternative assays. 

## 2. Methodology

This putative adverse outcome pathway (AOP) for lung fibrosis was developed based on the guidelines outlined by the Organization for Economic Cooperation and Development (OECD) [22]. To develop this AOP, we conducted an extensive literature search and review on the mechanisms of lung fibrosis, particularly on oxidative stress-related aspects of lung fibrosis and the effects of smoking on lung health. The extensive scientific knowledge is summarized as a framework in a simple, clear, and linear format. This AOP framework is expected to serve as an open, living entity and a foundational tool for the advancement of alternative assays and a better understanding of different events that drive lung fibrosis.

Literature search: A comprehensive and systematic search of the available literature was conducted using public literature databases, such as PubMed, as well as private scientific platforms like Web of Science. The search included all relevant articles without specifying any time limitations to ensure the incorporation of the most recent and up-to-date information. By encompassing the existing literature, we aimed to capture a broad spectrum of studies related to topics of interest up to the present date. Because the scientific literature is extensive on this topic, we cited only select original publications and reviews as appropriate. Several key word combinations were used for the literature search (Supplemental Table S1).

Inclusion and exclusion criteria: To ensure the relevance and reliability of the studies included in the review, we applied the following inclusion and exclusion criteria:

Inclusion criteria: (1) Most recent experimental articles as a priority for analysis; (2) Studies focusing on the relationship between oxidative stress and lung fibrosis; (3) Research related to the adverse effects of cigarette smoking, environmental toxins, and other stressors on lung fibrosis; (4) Studies describing the molecular mechanisms and pathways involved in oxidative stress-induced lung fibrosis, and (5) Research on adverse outcome pathways, particularly those related to lung fibrosis.

Exclusion criteria: (1) Studies unrelated to oxidative stress and lung fibrosis (2) Research lacking relevance to the proposed adverse outcome pathway for lung fibrosis, and; (3) Non-scientific articles, editorials, opinions, and case reports.

Data extraction and analysis: After we had conducted the literature search and applied the inclusion and exclusion criteria, relevant data were extracted from the selected articles. The information gathered was analyzed to identify key events and causal relationships related to oxidative stress-induced lung fibrosis. 

## 3. KEs and Mechanisms

**Figure 1:**
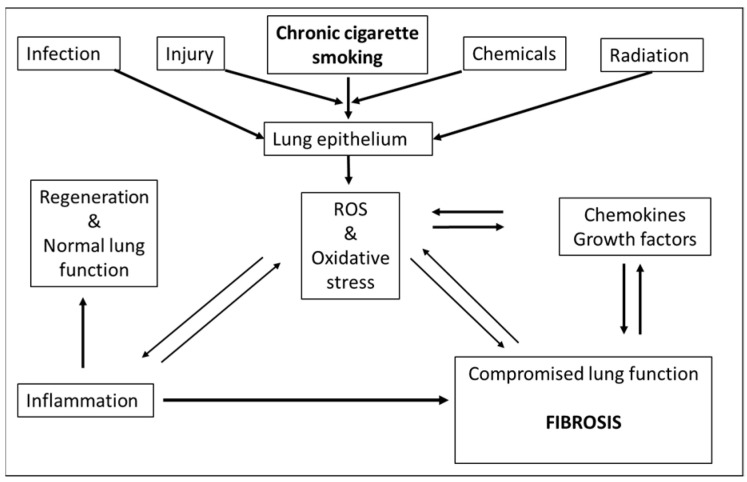
Feed-forward and feed-back events where ROS can contribute to fibrosis: Chronic cigarette smoking, infection, injury, toxic chemicals, drugs as well as radiation (UV, ionizing) may lead to formation of ROS. As a consequence, ROS adversely impact lung epithelium and may directly contribute to fibrosis or indirectly via enhanced inflammation. Fibrosis itself may feedback to ROS formation or foster generation of cytokines and growth factors which also can contribute to generation of ROS. Under normal circumstances (non-fibrotic response), the transient induction of inflammation by ROS is followed by tissue regeneration and restoration of normal lung function ([35], adapted and reprinted with permission).

Lung epithelial cells form a continuous lining of a pseudo-stratified cell layer along the inner walls of airways and form the first line of the defensive barrier for protection from environmental insults and microbial infections [1,2,32]. Various cell types, including basal, ciliated, goblet and alveolar epithelial cells, constitute lung epithelium, and the cellular structure varies considerably depending on their locations within the lung. The integrity and barrier function of lung epithelial cells is maintained by tight junctions which provide strength and regulate permeability. Lung epithelial cells, in a tightly regulated process, sense pathogens and injury, produce cytokines, recruit immune cells and undergo repair under normal conditions [32,33,34]. However, under pathological conditions, the repair process is dysregulated (Figure 1). For example, due to persistent injury or infection, lung epithelial cells can undergo apoptosis or phenotypic transformation to fibroblasts and contribute to inflammation and fibrosis. Such a situation may occur in chronic smoking, leading to lung fibrosis in susceptible individuals [16,32,33].

Thus, multiple cell types (including lung epithelial cells, immune cells and fibroblasts) could contribute to the dysregulated repair reported in lung fibrosis. Numerous altered intracellular signaling pathways, due to repeated injury to the lung epithelium, drive profibrotic processes that culminate in fibrosis and compromise lung function [2,7]. These complex and interdependent biological pathways of lung fibrosis are summarized in Supplemental Figure S1.

**Figure 2:**
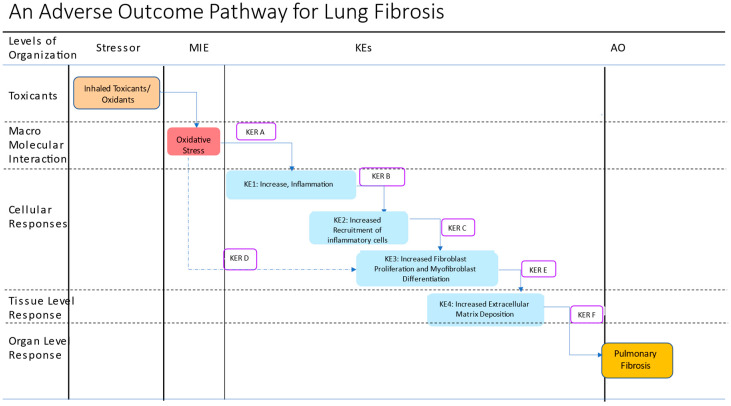
Schematic representation of a putative adverse outcome pathway for lung fibrosis. The putative AOP proposes oxidative stress as a MIE and consists of 4 KEs leading to the AO, lung fibrosis. A new KER, KER D is proposed to capture the direct effects of oxidative stress on KE3, primarily on TGF-1-mediated signaling. KER A: Increased oxidative stress leads to increased secretion of proinflammatory and profibrotic mediators; KER B: Increased proinflammatory mediators lead to increased recruitment of inflammatory cells; KER C: Increased recruitment of inflammatory cells leads to increased production of ROS and latent TFGF-beta-1 activation; KER D: Increased oxidative stress leads to increased fibroblast proliferation and myoblast differentiation; KER E: Increased cellular proliferation and differentiation lead to increased extracellular matrix deposition; KER F: Increased extracellular matrix deposition leads to pulmonary fibrosis. Different colored boxes are used to show various events: stressors, brown box; MIE, red box, KEs, blue, KERs open box, and the AO, dark yellow.

We propose an AOP for lung fibrosis that envisions oxidative stress-induced inflammation as the early molecular mechanism leading to downstream profibrotic cellular mechanisms. Based on the weight of evidence that we gathered, oxidative stress was considered as a MIE that leads to increased secretion of proinflammatory and profibrotic mediators (KE1). At the cellular level, a proinflammatory response induces recruitment of inflammatory cells (KE2), which in turn increase fibroblast/myofibroblast differentiation (KE3). At the tissue level, activated fibroblasts and myofibroblasts secrete and deposit extracellular matrix proteins (KE4). This process culminates in lung fibrosis as the adverse outcome. An overview of the main processes and pathways of this AOP is presented in Figure 2.

## 4. Cigarette Smoke, the Representative Stressor 

Exposure to stressors, such as CS, asbestos fibers, silica particles, carbon nanotubes or radiation, perturb the oxidant–anti-oxidant balance, overwhelm anti-oxidant defenses, and have been reported to promote fibrosis in the lung [9,13,36]. Here, we have used CS as an example of a stressor that induces oxidative stress [5,8,19,37,38]. CS is a complex, dynamic aerosol that is a mixture of several classes of chemicals and contains a large number of toxicants generated during the combustion process. The US FDA has identified 93 toxicants as hazardous and potentially hazardous compounds in CS and designated them as causative agents for several smoking-related diseases [39]. CS is rich in free radicals such as nitric oxide and contains quinones and semiquinones which are capable of inducing oxidation and reduction. In addition, CS contains many toxicants including volatile organic compounds (e.g., acrolein, crotonaldehyde and formaldehyde), polycyclic aromatic hydrocarbons and heavy metals (e.g., cadmium) that can cause oxidative stress [10]. At present, CS is described as stressor # 645 in the AOP knowledge base that impacts lung function, and we use the same descriptor. 

## 5. Oxidative Stress, the Molecular Initiating Event (MIE)

CS contains high concentrations of oxidants, and the local (lung) and systemic consequences of smoking-induced oxidative stress are widely understood [10]. Airway epithelium, which functions as a physiological barrier to inhaled harmful chemicals, is the primary target of smoking-induced oxidative stress [2,33]. Epithelial injury drives the production of endogenous reactive oxygen and nitrogen species (ROS and RNS) in airway epithelial cells and macrophages [40,41,42,43]. One of the early responses to CS exposure in airway epithelial cells (and several other cell types) is the rapid upregulation of the expression of oxidative stress response genes including, *HMOX1*, *GPX2*, *GCLC*, *GCLM*, *PRDX1* and *NQO1*, among others [44,45]. 

Oxidative stress depletes anti-oxidant defenses (e.g., glutathione) in smokers and the expression of the key antioxidant transcription factor Nrf-2 is downregulated [13,21,46]. As a result of these changes, biomarkers of oxidative stress, such as F2-isoprostane and other arachidonic acid metabolites and systemic inflammation markers such as white blood cells, are elevated in chronic smokers [47]. 

In vitro exposure of lung epithelial cell cultures to CS depletes intracellular glutathione levels [48]. Additionally, the ROS-activated transforming growth factor (TGF)-beta 1 signaling also suppresses Nrf2 expression. The downregulation of Nrf2 activity is reflected in a decreased expression of antioxidant enzymes such as catalase, superoxide dismutase 1 (SOD1) and 2 (SOD2), heme oxygenase 1, γ-glutamylcysteine synthetase and glutathione peroxidase 1 (GPX1), which further exacerbates oxidative stress [49,50,51,52]. A recent review summarized the extensive evidence on oxidative stress in various models of bronchial epithelial cells from exposure to CS preparations [53].

In a bleomycin mouse model of lung fibrosis, increased oxidative stress was evident from the downregulation of Nrf-2, which also resulted in the proliferation of lung fibroblast and the secretion of collagen [54]. Restoring the redox balance by treatment with activators of Nrf-2, such as dimethyl fumarate and sulforaphane, mitigated features associated with fibrosis in mice treated with bleomycin [55,56]. Oxidative stress is designated as an MIE in three other AOPs (#s 411, 424 and 425) as shown in Table 1, and due to the similarity of the underlying molecular events, we retain the same event id #1392 as the MIE for this AOP.

## 6. Increased Inflammation, KE1

A breach of the lung barrier function from exposure to inhaled toxicants results in increased epithelial permeability and cell death, and triggers inflammation by lung epithelial and the resident immune cells [33]. Oxidative stress triggers inflammatory responses in the lungs [57,58]. Exposure to CS induces the release of inflammatory cytokines from lung epithelial cells and immune cells [30,59,60]. 

For example, cultured bronchial epithelial cells, on exposure to CS extracts, release interleukin-8 (IL-8) which is a chemotactic factor for neutrophils [61]. Further, bronchoalveolar fluid (BAL) samples from smokers contain higher levels of IL-8 and increased counts of neutrophils [62]. Several other reports also indicate that the release/expression of proinflammatory mediators (such as tumor necrosis factor-alpha (TNF-*alpha*), chemokine ligands GRO-1 and CCL-2, and multiple interleukins) is secreted from alveolar epithelial cells and lung macrophages in response to oxidative stress arising from lung injury (KE1) [34,63,64,65,66,67,68,69]. 

Two similar KEs (event ids #149 and #901) which describe inflammatory responses exist in the AOP knowledge base. In the context of this AOP, the event id# 149, “Increase, Inflammation” is proposed as the KE 1 as it is more appropriate because of the context of inflammation following lung epithelial injury.

## 7. Increased Recruitment of Inflammatory Cells, KE2

Following lung injury, immune cells are recruited to the lung, and those cells produce mediators of inflammation, including ROS and proinflammatory cytokines [70,71,72]. Treatment with CS extracts releases the chemotactic proteins ICAM1, MCP, GM-CSF from lung epithelial cells [42,73,74,75,76]; this promotes further recruitment and activation of pro-inflammatory cells (KE2). Treatment of NHBE cells with CS preparations results in alterations in cytokines and the associated pathways, together with changes in xenobiotic and oxidative stress pathways [77,78]. Additionally, increases in the infiltration of neutrophils, dendritic cells, and lymphocytes were demonstrated in animal models of lung inflammation, fibrosis, emphysema, and CS-exposed in vitro systems [72,79,80,81,82]. While KEs #901 and 1497 address mechanisms of recruitment of inflammatory cells, because of its relevance to the AO, the KE #1497 is used in the current AOP as the KE2.

## 8. Increased Fibroblast Proliferation and Myofibroblast Differentiation, KE3

TGF-*beta* 1 pathway is widely recognized as the main inductor and regulator of fibrosis. TGF-beta 1 plays a central role in fibrotic processes in the lung by inducing epithelial–mesenchymal transition (EMT), driving the proliferation of fibroblasts and differentiation towards myofibroblasts (KE3) [32,35,83,84]. An existing KE in the AOP knowledge base captures the cellular events in the KE3, and the KE #1500 is proposed as the KE3.

## 9. Increased Extracellular Matrix Deposition (Accumulation of Collagen), KE4

Lung fibrosis is characterized by excessive deposition of ECM (KE4) in the lung parenchyma by different stressors [60,85,86,87]. The abnormal ECM deposition remodels the normal lung tissue architecture by replacing epithelial tissue with fibroblasts, compromising gas exchange and leading to respiratory failure and death [88]. Multiple stressors and increased oxidative stress can additionally induce the release of TGF-beta 1 and collagen secretion from human lung fibroblasts via cellular influx through chloride channels. Activated TGF-beta 1, in turn, induces the expression of ECM proteins such as fibronectin, collagen I, collagen III, α-smooth muscle actin (ACTA2), vimentin and MMP-9 from fibroblasts [84,89,90,91,92], thereby leading to the excessive deposition of ECM [13]. TGF-beta 1 also induces the secretion of plasminogen activator-1, which blocks plasminogen activation, fibronectin proteolysis, and fibroblast apoptosis [93]. Additionally, TGF-beta 1 further promotes the remodeling of ECM and EMT in ROS-dependent mechanisms [94]. The KE #68 in the AOP knowledge base captures these tissue-level events.

In summary, inhaled toxicants/oxidants drive lung fibrosis (AO) by creating persistent oxidative stress (MIE) and the subsequent secretion of proinflammatory and mediators (KE1), which feed on each other. Unresolved proinflammatory response induces the recruitment of inflammatory cells (KE2) and establishes a state of chronic inflammation, which in turn promotes fibroblast proliferation and myofibroblast differentiation (KE3). This chronic inflammation causes recurrent lung injury, abnormal wound healing, and fibrosis in susceptible individuals. At the tissue level, activated myofibroblasts deposit increased ECM proteins (KE4) and subsequently lead to lung fibrosis as the AO (Figure 2). We will use an existing AO (event id #1458) shared by AOPs 173, 241 and 347 as the AO in this AOP for lung fibrosis. 

## 10. Key Event Relationships

It is well established that oxidative stress and inflammation co-exist and constitute feedback loops. Substantial knowledge exists on the role of persistent oxidative stress promoting proinflammatory profibrotic changes in the lung. In this AOP, we propose that oxidative stress, as an MIE, causes lung injury (KE1) and drives the subsequent KEs (2–4) and the AO. The relationship between inhaled toxicants (e.g., cigarette smoking) oxidative stress and inflammation through KERs A and B, is amply demonstrated. Similarly, the role of profibrotic mediators (KER C) and ECM deposition (KER E) to the AO are well described in the AOP 173 [25]. These KERs are not discussed further in this manuscript. Here we propose a new KER, KER D in which the MIE directly leads to events in the KE #3 (Increased fibroblast proliferation and myofibroblast differentiation) through a new KER, KER D (Figure 2) [30]. 

## 11. MIE → KE3 Increased Oxidative Stress Leads to Increased Fibroblast Proliferation and Myoblast Differentiation, KER D

Lung fibroblasts play an important role in the remodeling of the extracellular matrix and subsequent progression to lung fibrosis [95]. Exposure of CS extracts induces senescence in lung fibroblasts, and subpopulations of senescence-resistant fibroblasts have been shown to undergo increased proliferation, mobility and secretion of TGF beta-1. Treatment with antioxidants blocked CS extract-induced proliferation of senescence-resistant fibroblasts, but not the other responses [96]. 

TGF beta-1 is an important multifunctional cytokine which regulates fibrosis and is tightly regulated through multiple mechanisms, including ROS [84]. Several reports indicate that ROS directly activates TGF beta 1 and induces its release from macrophages and airway epithelial cells [21,97], and thus regulates TGF-beta 1-induced profibrotic effects [98]. 

TGF beta-1 is synthesized and secreted into the extracellular space as a large latent complex containing mature dimers of TGF beta-1 bound to latency-associated peptide (LAP). One of the key mechanisms of activation of TGF beta-1 involves its release from LAP, through a redox mechanism [99]. Nitrosative stress was also reported to interfere with the ability of LAP to bind and inactivate TGF beta-1 [100]. ROS generated from ionizing radiation have been shown to release TGF-beta 1 from the latent form in an isoform-specific manner. The latent form of TGF beta 1 was hypothesized to contain a redox switch, and the oxidative modification of LAP-beta 1 was shown to activate TGF beta-1 [101]. Similarly, asbestos-derived ROS activate TGF beta-1, which was blocked by antioxidant enzymes (SOD and catalase), deferoxamine (chelates iron), or anti-oxidants [102,103]. 

Separately, TGF beta-1 itself was reported to induce ROS production and regulate the expression of anti-oxidant and pro-oxidant genes leading to further exacerbation of oxidative stress [13,104,105]. The anti-oxidant N-acetyl cysteine (NAC) reversed several aspects of TGF beta 1-induced EMT in rat alveolar epithelial cells, including restoration of intracellular glutathione levels and cellular morphology. The treatment with NAC also reduced the levels of TGF beta 1-mediated increases in mesenchymal markers such as α-smooth muscle actin and vimentin [94]. It was also reported that continuous treatment with aqueous extracts of CS induced the expression of TGF beta-1 and resulted in phenotypic changes consistent with fibroblasts [106]. 

Findings from murine models also lend support to the role of oxidative stress in lung fibrosis. A recent study indicates that the ROS-Nrf2 pathway mediates the development of TGF beta-1-induced EMT through the activation of Notch signaling, although its role in fibrosis remains unknown at present [107]. Furthermore, in a knockout-mouse model (deficient in the p47 subunit of NADPH oxidase) BAL cells were unable to produce ROS in response to bleomycin treatment. It was observed that in the bleomycin-treated group, collagen deposition was absent in the lungs, indicating the importance of endogenous ROS in lung fibrosis [108]. 

Additional evidence for ROS as drivers of lung fibrosis comes from the bleomycin-induced mouse models. First, inhibition of bleomycin-induced oxidative stress with anti-oxidant treatments suppressed increases in inflammatory cell counts and blocked lung fibrosis. The authors measured the levels of malondialdehyde (MDA), extracellular SOD, total antioxidant capacity and ROS in lung tissue and of NF-kB-mediated inflammatory genes expression of TNF-*alpha*, IL-1 beta and IL-6 in these animal models [109,110]. Second, glutaredoxin, an enzyme involved in redox signaling, reverses a post-translational modification known as protein S-glutathiolation and restores cysteine residues on target proteins [111,112]. Recently, it was shown that mice deficient in the glutaredoxin gene (GLRX) show excessive collagen deposition following either treatment with bleomycin or recombinant TGF beta-1. Further, transgenic mice that conditionally overexpress GLRX suppressed TGF beta-1-induced lung fibrosis [113]. Thus, several lines of evidence support the role of ROS and oxidative stress in the development of lung fibrosis (KER D).

## 12. Application of the AOP

Multiple stressors, including CS, cause imbalance in oxidants and reductants, chronic inflammation, and fibrotic responses in the lung [36,114,115,116]. Additionally, lung fibrosis increases the risk of lung cancer [8]. With cigarette smoking being a significant risk factor, a focused AOP, such as this one, may aid in identifying and evaluating novel targets for early diagnosis and treatment options for lung fibrosis that are more efficacious. 

Combustion-related toxicants are major drivers of smoking-related diseases, which develop with a latency of several decades [10]. Emerging and traditional non-combusted tobacco products do not generate combustion-related toxicants. In addition, aerosols of the inhaled potentially reduced risk products are chemically less complex and contain significantly lower levels of oxidants [117], as well as not inducing oxidative stress-response genes [44]. Non-combustible products have been hypothesized to have a lower risk of smoking-related diseases [118,119]. This approach is consistent with the guidance issued by the US FDA that for tobacco products to gain approval through the FDA’s modified risk tobacco product review must demonstrate or be reasonably likely to result in reduced risk, and a reduced public health burden of cigarette smoking [120]. In that context, AOPs are suggested as potential tools to assess the relative risk of cigarettes and alternative tobacco products [121]. Thus, this AOP should guide the development of fit-for-purpose alternative assays for the evaluation of candidate reduced risk tobacco products.

It should be noted that smoking cessation continues to be the best option to reduce harm from smoking [122], although tobacco harm reduction could include complete substitution of smoking with less harmful tobacco products for those who are unwilling to quit [123]. 

## 13. Perspectives and Expectations

This putative AOP is proposed as an open framework for investigation of disease mechanisms of lung fibrosis, and potentially aid in regulatory evaluations of emerging candidate-modified risk tobacco products. The proposed AOP uses CS as a representative stressor, which is epidemiologically linked to the adverse outcome. The mechanistic relationships proposed herein may support the discovery and testing of treatment options for lung fibrosis. Like all AOPs, it integrates several types of evidence and allows for the development of specific assays and biomarkers based on the extensive scientific knowledge. 

There are some limitations to this putative AOP. For example, it is a theoretical framework and will need to go through the review process by the OECD, and the assays for selected key endpoints will require validation. Together with the development and continued standardization of whole CS aerosol exposure systems, the in vitro systems may be better suited for regulatory evaluations [124]. Second, this AOP is particularly focused on the role of inhaled oxidants as the stressors, and the role of genetic risk factors in lung fibrosis is beyond the scope of this manuscript [125]. Third, while CS is etiologically linked to lung fibrosis, it is a mixture of numerous chemicals and toxicants, and the targets of CS induced-oxidative stress could be multiple. CS, in addition to inducing a state of chronic oxidative stress, also causes genetic [10] and epigenetic changes [126]. However, careful evaluation of the consensus early effects of exposure to CS, such as depletion of anti-oxidants, induction of genes responsive to oxidative stress and markers of lung epithelial cell injury, could be useful in the application of this putative AOP for regulatory purposes. 

## 14. Discussion

Oxidative stress and inflammation are two key inter-related biological processes that are important for normal homeostasis, and their deregulation causes (or, is associated with) the development of many diseases. This AOP, supported by solid scientific evidence, integrates and organizes multiple well-established pathways of fibrosis (Supplemental Figure S1) into a tractable framework. 

Lung fibrosis is associated with significant disease burden and mortality by itself and as a comorbidity in lung cancer and COPD. Consensus mechanisms show that repetitive lung injury from persistent oxidative stress due to chronic smoking drives a cycle of oxidative stress and inflammation (KEs 1 and 2) [10]. Under these conditions, normal tissue repair is deregulated and becomes profibrotic, leading to the aberrant proliferation and differentiation (KE3), EMT and deposition of ECM (KE4).

The AOP knowledge base currently holds eight AOPs with lung fibrosis as an AO [25]. Among them, the AOP 173 is the most mature and advanced one that presents a critically reviewed framework for testing nanoparticles [127]. The AOP 173 lists bleomycin and carbon nanoparticles as stressors and ascribes robust inflammation as a central mechanism.

The remaining AOPs are more selective and are at various stages of development. AOPs #206 and #347 involve PPAR γ and TGFβ pathways, respectively, and limited information is available on these two AOPs. The AOP 241 also describes lung fibrosis as an AO with nanotubes as the stressor-involving TGFβ pathway. Two other related AOPs (#414 and #415) describe the activation of AHR as an MIE and progress through the TGFβ and IL6 pathways. Additionally, two AOPs, involving the angiotensinogen-converting enzyme (#319) and the inactivation of the angiotensinogen II type I receptor (#382), are listed. Whereas particulate matter PM 2.5 is one of the listed stressors for AOP #319, no stressors are provided for AOP #382 [25].

The etiology of IPF is unknown, although several risk factors have been identified. Besides cigarette smoking, other agents, such as environmental exposures (nanoparticles, fumes, and other particulate matter), viral infections and comorbidities (diabetes mellitus, gastroesophageal disease, and obstructive sleep apnea) have been identified as risk factors for lung fibrosis [9,128]. While many cellular mechanisms such as cellular senescence and apoptosis contribute to lung fibrosis, this AOP is based on the known epidemiological linkage to CS as a model stressor and its established mechanism of oxidative stress as the MIE. 

The current putative AOP leverages existing events described in the AOP knowledge base and utilizes them to describe the effects of stressors, starting from an MIE through the AO (Table 1). Oxidative stress has been used as an MIE in three related AOPs (#411, #424 and #425), whereas the KE1 is shared with AOP #149. The remaining KEs and the AO are shared with AOP #173. In building this AOP, we formally assigned a new KER, KER D, to recognize a direct role of the oxidative stress in increasing fibroblast proliferation and differentiation myofibroblasts (KE #3). Clearly, an imbalance in redox status can arise from ROS in external toxicants or from deregulated internal cellular processes as well. In chronic smoking and exposure to other toxicants, both mechanisms could drive fibrotic changes in susceptible individuals.

In conclusion, we propose a simple and tractable framework of key events that promote lung fibrosis. This framework synthesizes key risk factors, initial injury to the pulmonary epithelium, tissue responses that mediate fibrosis, and distills the extensive knowledge into a testable model consisting of well-defined key events.

## Figures and Tables

**Table 1:** Summary of MIE and KEs shared with existing AOPs: AOPs are modular by concept and the modules (e.g., KEs) and other information can be shared across different AOPs to describe common mechanisms. The AOP knowledge base is a repository of AOPs and related information [25]. Various AOP-related entities, such as MIEs and KEs and AOPs, are designated with numbers. The putative AOP for lung fibrosis is assembled by utilizing an existing MIE and the KEs developed for other mechanistically related AOPs, as described in the text. The table lists the shared event id numbers (#sign) and the respective AOP numbers from the AOP knowledge base.

Event	Event Descriptor (Stressor/MIE/KE/AO)	Event Id #	Shared AOPs #
Stressors	Inhaled toxicants/oxidants, e.g., Cigarette smoke	645	411, 424, 425
MIE	Oxidative stress	1392	411, 424, 425
KE1	Increase, Inflammation	149	27. 115, 206, 280, 439
KE2	Increased recruitment of inflammatory cells	1497	173, 303, 392, 377, 451
KE3	Increased fibroblast proliferation and myofibroblast differentiation	1500	173
KE4	Increased extracellular matrix deposition (accumulation of collagen)	68	38, 241, 144, 319, 173
AO	Lung fibrosis	1458	173, 241, 347

## Data Availability

Not applicable.

## Supplementary Materials

The supporting information can be downloaded at: https://www.mdpi.com/article/10.3390/ijms241512490/s1.
