# Supplementary material for: Oxidative Stress and Lung Fibrosis: Towards an Adverse Outcome Pathway
Source: Int J Mol Sci. 2023 Aug 6;24(15):12490. doi: 10.3390/ijms241512490 (PMC10419527; doi:10.3390/ijms241512490)
Supplement: Supplementary file 1 [file ijms-24-12490-s001.zip › ijms-2509296-supplementary.pdf]

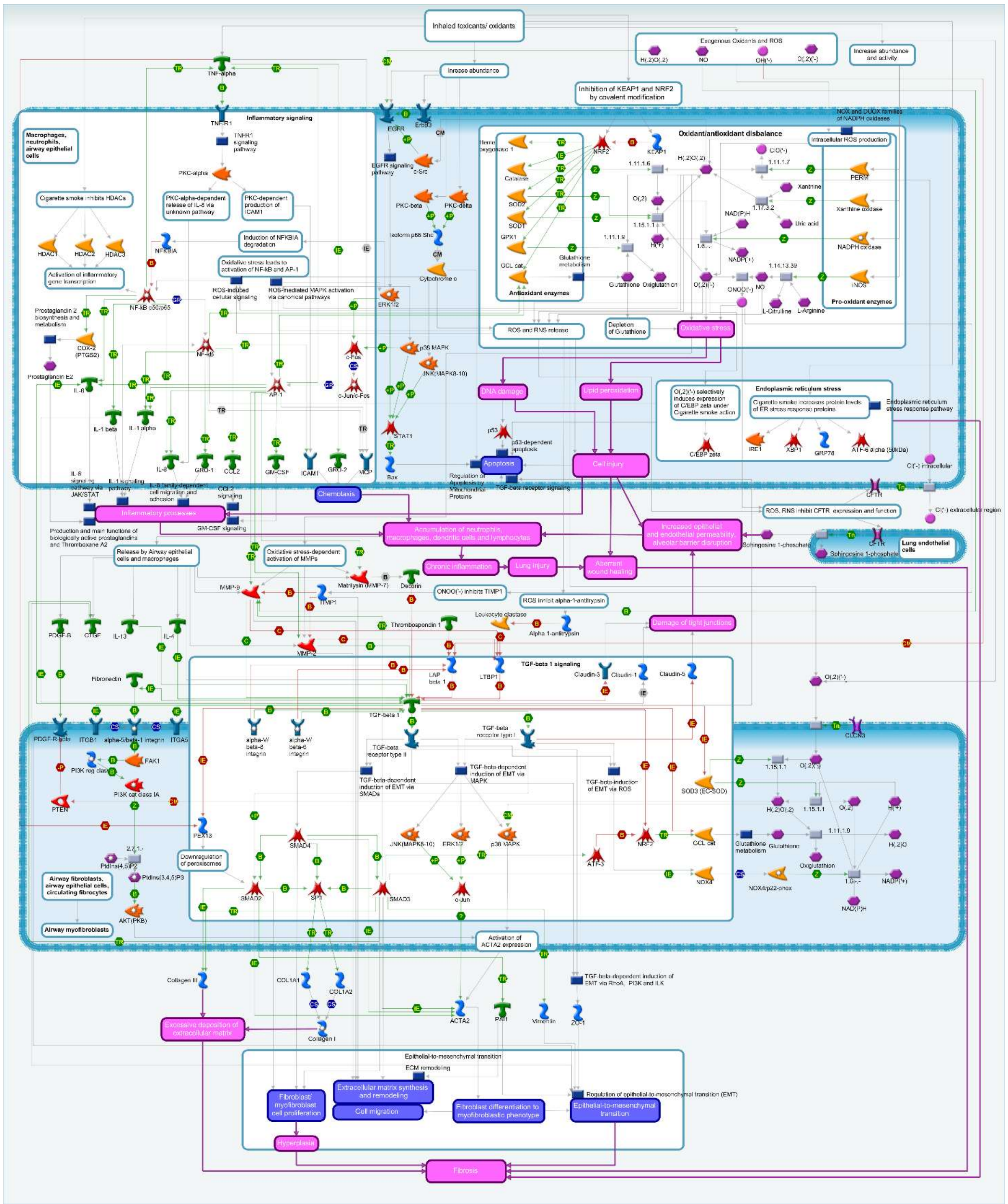

Supplemental Figure S1. Mechanisms that contribute to lung fibrosis. Lung fibrosis is characterized by injury and loss of lung epithelial cells, inflammation, abnormal wound healing, accumulation of fibroblasts/myofibroblasts and aberrant remodeling of the lung parenchyma. Certain conditions, such as chronic cigarette smoking and exposure to different toxicants, induce oxidant/antioxidant disbalance, suppression of anti-oxidant enzymes and increased generation of endogenous ROS and RNS leading to oxidative stress. ROS and RNS activate intracellular signaling pathways leading to DNA damage, cell injury, apoptosis and activation of transcription factors, such as NF-kB and AP-1 in airway epithelial cells and macrophages. NF-kB and AP-1 stimulate expression of inflammatory genes, promoting accumulation of neutrophils, macrophages, dendritic cells and lymphocytes. Chronic lung inflammation leads to unresolved lung injury. The recurrent injury leads to permanent fibrosis, through a process that is considered to represent a form of abnormal wound healing. Activated macrophages release pro-fibrotic factors such as TGF *beta*-1, IL-4, IL-13 and others, which promote additional ROS generation, EMT, ECM synthesis and remodeling and fibroblast-to-myofibroblast differentiation driving the progression to lung fibrosis. The Supplementary Figure S1 was generated using MetaCore Pathway Map Creator tool Version 2.6.0 (Clarivate Analytics, Philadelphia PA).

Supplemental Table S1. Key word combinations used in the literature search to develop the AOP for lung fibrosis.

oxidative stress	lung fibrosis	cigarette smoking	environmental toxins
oxidative damage	pulmonary fibrosis	tobacco smoking	environmental pollutants
redox imbalance	fibrotic lung disease	smoking habit	toxic environmental agents
reactive oxygen/nitrogen species (ROS/RNS) toxicity	interstitial lung fibrosis	inhalation of tobacco smoke	harmful environmental substances
free radicals	pulmonary scarring	cigarette consumption	pollutants in the environment
oxygen radicals	lung tissue fibrosis	nicotine exposure	chemical environmental hazards
oxidative burden	pulmonary fibrogenesis	tobacco use	asbestos fibers
oxidative injury	inflammation		silica particles
glutathione	inflammatory response		carbon nanotubes (CNT)
antioxidant/oxidant disbalance	fibrotic response		radiation
superoxide radical	IPD (Idiopathic pulmonary fibrosis)		
hydrogen peroxide	COPD (Chronic obstructive pulmonary disease)		
hydroxyl radical			
peroxynitrite			
aldehydes			
